# A method for determining workers’ exposure concentration to airborne nitrilotriacetic acid

**DOI:** 10.1093/joccuh/uiaf025

**Published:** 2025-05-07

**Authors:** Ai Yamada, Kenta Ishii, Akito Takeuchi, Tomiko Tashiro, Ginji Endo, Mariko Ono-Ogasawara

**Affiliations:** Osaka Occupational Health Service Center, Japan Industrial Safety and Health Association, 2-3-8 Tosabori, Nishi-Ku, Osaka 550-0001, Japan; Department of Pathophysiological Laboratory Sciences, Nagoya University Graduate School of Medicine, 1-1-20 Daikominami, Higashi-ku, Nagoya, Aichi 461-8673, Japan; Technical Support Department, Japan Industrial Safety and Health Association, 5-35-2 Shiba, Minato-Ku, Tokyo 108-0014, Japan; Occupational Health Research and Development Center, Japan Industrial Safety and Health Association, 5-35-2 Shiba, Minato-Ku, Tokyo 108-0014, Japan; Laboratory of Environmental Toxicology and Carcinogenesis, School of Pharmacy, Nihon University, 7-7-1 Narashinodai, Funabashi, Chiba 274-8555, Japan; Osaka Occupational Health Service Center, Japan Industrial Safety and Health Association, 2-3-8 Tosabori, Nishi-Ku, Osaka 550-0001, Japan; Osaka Occupational Health Service Center, Japan Industrial Safety and Health Association, 2-3-8 Tosabori, Nishi-Ku, Osaka 550-0001, Japan; Osaka Occupational Health Service Center, Japan Industrial Safety and Health Association, 2-3-8 Tosabori, Nishi-Ku, Osaka 550-0001, Japan; National Institute of Occupational Safety and Health, 6-21-1 Nagao, Tama Ward, Kawasaki City, Kanagawa Prefecture 214-8585, Japan

**Keywords:** air sampling method, gas chromatography–mass spectrometry, nitrilotriacetic acid, personal exposure measurement, workplace air

## Abstract

**Objectives:**

This study aimed to establish a method to determine workers’ exposure concentrations to airborne nitrilotriacetic acid (NTA).

**Methods:**

The sampler used an air sampling cassette containing a glass-fiber filter (GFF). After sampling, NTA extracted from the GFF using ultrapure water was derivatized using a boron trifluoride-methanol complex methanol solution and analyzed using a gas chromatograph–mass spectrometer. The developed method was validated for the following parameters: retention efficiency, storage stability, method quantitation limit, and reproducibility.

**Results:**

The retention efficiency for NTA on the GFF was 94%-101%, with the relative SD indicating the overall reproducibility (range 0.9%-2.4%). NTA on the GFF was stable at 4°C for at least 7 days. The method quantitation limit was 4.8 μg per sample.

**Conclusions:**

The developed method will be useful for risk assessments because it can determine workers’ exposure concentrations to NTA ranging from 0.02 to 4 mg/m^3^ in a 240 L sampling volume; the quantitation limit is 4.8 μg per sample.

## Teaser

Key points

**What is already known on this topic:** Nitrilotriacetic acid (NTA) is used in a wide range of industrial applications. The Deutsche Forschungsgemeinschaft has proposed occupational exposure limits based on the toxicity of NTA, but no method existed to measure airborne NTA exposure concentrations for workers.

**What this study adds:** This study presents a method for collecting NTA using a glass-fiber filter and analyzing it using a gas chromatograph–mass spectrometer. The developed method can measure workers’ exposure concentrations to NTA ranging from 0.02 to 4 mg/m^3^ in a 4-hour sampling period.

**How this study might affect research, practice, or policy:** The established approach can estimate workers’ NTA exposure levels and subsequently help prevent their exposure to NTA.

## Introduction

Nitrilotriacetic acid (NTA; CAS Registry Number: 139-13-9) is used as a chelating agent, a builder in synthetic detergents, an eluting agent to purify rare earth elements, and a boiler feed water additive. It is also used in water and textile treatment, metal plating and cleaning, and pulp and paper processing.^1^ From 2019 to 2021, NTA was chosen as a target chemical for a workplace chemical risk assessment project by the Japanese Ministry of Health, Labour and Welfare (MHLW) owing to its classification as a Group 2B compound (possibly carcinogenic to humans) by the International Agency for Research on Cancer^1^ and the Japan Society for Occupational Health (JSOH).^2^ An occupational exposure limit (2 mg/m^3^) for NTA has been proposed by the Deutsche Forschungsgemeinschaft (DFG)^3^ (as acid, avoid simultaneous exposure to iron compounds [formation of FeNTA]) (Maximale Arbeitsplatzkonzentrationen, MAK); however, no such limits have been proposed by the JSOH or the American Conference of Governmental Industrial Hygienists. Our literature survey revealed no existing method for determining workers’ exposure concentration to airborne NTA; therefore, this study aimed to develop one.

## Materials and methods

### Materials

NTA was purchased from Dojindo Laboratories. NTA-d_9_ was purchased from C/D/N Isotopes Inc. and used as an internal standard (IS). Boron trifluoride-methanol complex (BF_3_/MeOH) methanol solution and 2,2-dimethoxypropane (DMP) were sourced from FUJIFILM Wako Pure Chemical Corporation and Tokyo Chemical Industry, respectively. All other reagents were above analytical grade. Ultrapure water produced by a PURELAB flex-3 (Organo Corp.) was used. Phosphate buffer solution (1 mM) was made by dissolving potassium dihydrogen phosphate in ultrapure water and adding 50% sodium hydroxide solution to adjust the pH to 7.0. Standard stock solution (800 μg/mL) and IS solution (1 mg/mL) were prepared in ultrapure water with the help of sonication (4 hours).

### Analytical procedure and instrumental conditions

The analysis of NTA was carried out according to the method for determining NTA in water reported by Nishikawa and Okumura,^4^ with minor modifications. After sampling, the glass-fiber filter (GFF) was placed in a polypropylene test tube. Ultrapure water (10 mL) and IS solution (100 μL) were added to the tube, which was vortex-mixed (10 minutes) and then centrifuged (1870 × *g*, 10 minutes). A supernatant aliquot (100 μL) was transferred to a glass test tube. Hydrochloric acid (35.0%-37.0%, 10 μL) and DMP (1 mL) were added to the tube, which was vortex-mixed (10 seconds) and then left to remain (room temperature, 10 minutes). The sample was evaporated to dryness (80°C, 15 minutes) using an aluminum block bath (Dry Thermo Unit DTU-2C, TAITEC Co.) under a stream of nitrogen. BF_3_/MeOH solution (1 mL) was added to the tube, which was capped and vortex-mixed (10 seconds) and then heated (80°C, 30 minutes) in the aluminum block bath. After cooling to room temperature, phosphate buffer solution (1 mL) and dichloromethane (2 mL) were added to the tube, which was vortex-mixed (10 seconds) and then centrifuged (1870 × *g*, 10 minutes). The lower dichloromethane layer (1 mL) was transferred to a fresh glass test tube, dehydrated by adding anhydrous sodium sulfate (1.5 g), centrifuged (1870 × *g*, 10 minutes), and then transferred to an autosampler vial for analysis. The samples were analyzed using a gas chromatograph–mass spectrometer (GC–MS; 7890B-5977B HES; Agilent Technologies) equipped with a DB-35 ms capillary column (30 m × 0.25 mm ID, film thickness 0.25 μm; Agilent Technologies). The carrier gas (helium) flow rate was 1.0 mL/min. The operating conditions for GC were as follows: inlet temperature at 250°C; injection volume 1 μL; pulsed split mode with a 50:1 split ratio and a pulse pressure of 25 psi (172.4 kPa) for 1 minute; column temperature at 70°C for 1 minute and then increased to 280°C at 10°C/min; transfer line temperature at 280°C. The operating conditions for MS were as follows: quadrupole temperature at 150°C; ion source temperature at 280°C; electron ionization mode with electron energy of 70 eV. Synchronous selective ion monitoring (SIM)/scan mode was used to acquire SIM and scan data in a single run. The trimethyl derivatives of NTA (NTA-tri-Me) and IS (IS-tri-Me) were identified by confirming the mass fragmentation obtained from scan data acquired in the range of *m*/*z* 40 to 250. The quantifier and qualifier ions used for the SIM were *m*/*z* 174 and 233 for NTA-tri-Me, and *m*/*z* 180 and 239 for IS-tri-Me, respectively.

### Validation procedures for sampling and storage methods

The sampler used an air sampling cassette (catalog no. 225-3LF; SKC Inc., Eighty Four, PA, USA) containing a GFF punched to 37 mm in diameter (catalog no. AP 2004200; Merck Millipore Ltd, Darmstadt, Germany). Method validation was performed according to the MHLW guideline.^5^ This guideline states that the minimum sampling time required to measure workers’ personal exposure concentrations to chemical substances is 4 hours. Therefore, the time for passing room air through the GFF was set to 4 hours for the retention efficiency and storage stability tests. The procedures for these tests were as follows: 2 levels of spiking NTA standard solutions (80 and 800 μg/mL) were prepared in ultrapure water. The GFF was spiked with either 60 μL of 80 μg/mL NTA standard solution, or 60, 600, or 1200 μL of 800 μg/mL NTA standard solution. After leaving the NTA-spiked GFF at room temperature to dry completely, it was set into the air sampling cassette, and then room air (temperature 22.7°-24.1°C; relative humidity 25%-48%) was drawn into the cassette using an AirChek 2000 sampling pump (catalog no. 210-2002; SKC Inc.) set at a flow rate of 1 L/min for 4 hours. For the storage stability test, the cassettes passing room air were sealed without removing the GFF and refrigerated at 4°C for 7 days. For these tests, the NTA-spiked amounts were 4.8, 48, 480, and 960 μg corresponding to approximately 0.02, 0.2, 2, and 4 mg/m^3^, respectively, representing approximately 0.01, 0.1, 1, and 2 times the DFG-proposed MAK values.^3^ To obtain a calibration curve, the standard solutions of NTA were prepared from the standard stock solution by dilution with ultrapure water at 8 concentrations, namely, 0.48, 2.4, 4.8, 12, 24, 48, 72, and 96 μg/L. These samples were prepared and analyzed using the aforementioned procedure. Calibration curves were constructed by plotting the peak area ratio of the NTA-tri-Me to the IS-tri-Me versus their respective concentrations.

**Table 1:** Retention efficiency and storage stability tests^a^

**Spiked amount,** ^**b**^ **μg**	**Retention efficiency, % (*n* = 5)**		**Storage rate, % (*n* = 3)**				
			**O days**	**1 day**	**3 days**	**5 days**	**7 days**
	**Mean ± SD**	**RSD**	**%**	**%**	**%**	**%**	**%**
4.80	94 ± 2.3	2.4	95, 100, 106	96, 102, 106	92, 99, 107	101, 115, 116	102, 106, 106
48.0	100 ± 1.5	1.6	99, 100, 101	100, 100, 101	98, 99, 100	99, 101, 102	102, 104, 105
480	100 ± 0.9	0.9	100, 100, 100	101, 102, 103	100, 102, 103	100, 101, 102	103, 103, 106

Abbreviation: RSD, relative standard deviation.

a The glass-fiber filters (GFFs) spiked with nitrilotriacetic acid were set into the air sampling cassette, and then room air (temperature 22.7-24.1°C; relative humidity 25%-48%) was drawn into these cassettes using the sampling pump at a flow rate of 1 L/min for 4h. For the storage stability test, the cassettes passing room air were sealed without removing the GFF from them and refrigerated at 4°C for 7 days.

b The spiked amounts correspond to air concentrations of approximately 0.02-4 mg/m^3^.

**Figure 1:**
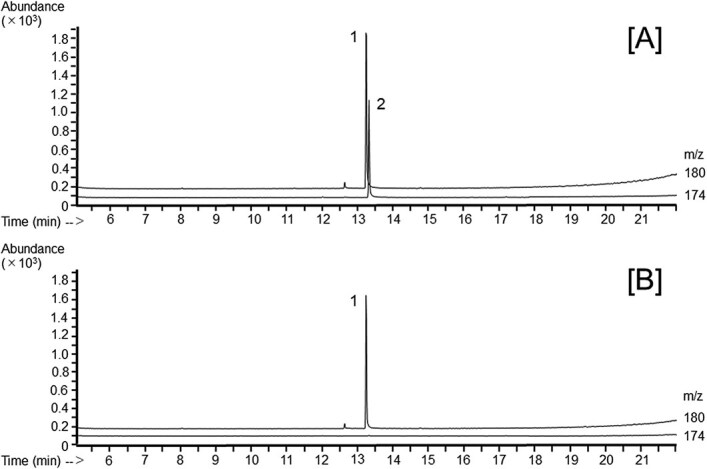
Typical reconstructed mass chromatograms of the glass-fiber filters with 240 L of room air passing through them after being spiked with (A) and without (B) 48 μg of nitrilotriacetic acid (NTA). The peaks of the trimethyl derivatives of (1) nitrilotriacetic acid-d_9_ (internal standard, IS-tri-me) and (2) nitrilotriacetic acid (NTA-tri-me) are indicated.

## Results

The validation results of the developed method are as follows. The retention efficiency of NTA on the GFF, including the extraction efficiency of NTA from the GFF, was 94%-101%, with the relative SD ranging from 0.9% to 2.4% (Table 1). These results indicate that the GFF is an appropriate collection medium for sampling NTA in workplace air and that the proposed method is highly reproducible and reliable. NTA storage rates on the GFF, calculated by comparing the amount of NTA remaining on the GFF after storage with those analyzed before storage, showed 95%-106% after 0 days, 96%-106% after 1 day, 92%-107% after 3 days, 99%-116% after 5 days, and 102%-107% after 7 days of storage in a refrigerator at 4°C (Table 1). The calibration curve showed linearity from 0.48 to 96 μg/mL, with a coefficient of determination (*R*^2^) of 0.999. The instrumental quantitation limit was 0.17 μg per sample, calculated as 10 times the SD (*n* = 5) of the peak area ratio of the lowest standard (0.48 μg/mL) divided by the calibration curves slope. The method quantitation limit was 4.8 μg per sample, established as the least NTA amount that resulted in >90% retention efficiency within the studied range.

## Discussion

The following were examined in this study: (1) collection medium and extraction solution and (2) analytical method and sample preparation procedure. Because NTA is solid with a very low vapor pressure at ambient temperature, it is presumed to be aerosolized in workplace air. Therefore, we adopted the GFF as a collection medium. Next, we explored a suitable solvent for extracting NTA from the GFF after sampling. Although NTA was insoluble in methanol, ethanol, tetrahydrofuran, ethyl acetate, 1,4-dioxane, chloroform, dichloromethane, and hexane, it was somewhat soluble in ultrapure water, with a maximum soluble concentration of approximately 1 mg/mL according to the literature.^6^ Therefore, ultrapure water was adopted as the extraction solution and used to prepare a standard stock solution.

There have been no reports of the determination method for NTA in the air (such as workplace air or atmosphere); however, several analytical methods have been published for determining NTA in water (including river water, wastewater, and tap water).^4^^,^^7–12^ These methods used GC with flame ionization detection^7^ or with MS,^4^^,^^8–10^ or high-performance liquid chromatography with ultraviolet detection^11^ or with fluorescence detection,^12^ and required a derivatization procedure suitable for these respective analytical approaches before analysis. In the reported GC methods, several derivatization procedures such as trimethylsilylation,^7^ iso-propyl esterification,^8^ methyl esterification,^4^^,^^10^  *n*-propyl esterification,^9^ and *n*-butyl esterification^9^ were used to transform NTA into highly volatile compounds to enable GC analyses. We adopted Nishikawa and Okumura’s^4^ GC–MS method using methyl esterification with BF_3_/MeOH solution with a few modifications (sample preparation procedure and IS substance). In their sample preparation procedure, the sample solution was evaporated to dryness with a rotary evaporator and a stream of nitrogen gas before derivatization with BF_3_/MeOH solution. Although the sample volume required in our sample preparation procedure was small, to remove water completely from the samples in a shorter time, we applied the sample preparation method with DMP used in our previous studies.^13^^,^^14^ DMP reacts with water in the presence of hydrogen ions to produce methanol and acetone. Therefore, DMP not only removes water but also facilitates subsequent evaporation to dryness. The proposed preparation procedure allows evaporation to dryness within 15 minutes. It was able to derivatize NTA and IS to NTA-tri-Me and IS-tri-Me, respectively. Our mass spectrum of NTA-tri-Me agreed with that of Nishikawa and Okumura.^4^ Typical reconstructed mass chromatograms of samples from the GFF spiked with an NTA standard solution and from a blank GFF are shown in Figure 1A and 1B, respectively. No NTA was detected from the blank GFF, indicating that the GFF used in this study did not contain NTA. Although the proposed method can measure workers’ NTA exposure concentrations up to 2 times the DFG-proposed MAK value with a sampling time of 4 hours, if the workers’ NTA exposure concentrations are expected to exceed that value in the preliminary assessment, or if the workers’ NTA exposure measurement results exceed that value, the measurement or re-measurement should be performed with a shorter sampling time.

## Conclusions

To our knowledge, we have successfully developed the first method for determining workers’ exposure concentrations to airborne NTA. The proposed method enables the measurement of workers’ NTA exposure concentrations from 0.02 to 4 mg/m^3^ in a 4-hour sampling period, corresponding to 0.01–2 times the DFG-proposed MAK values. Therefore, it will be useful for estimating workers’ exposure levels to NTA. A major limitation of this study was that the experiment used GFFs spiked with NTA standard solutions because continuous generation of standard aerosols at known concentrations was not feasible. This limitation is a common and significant issue in studies developing air sampling methods for particulate chemical substances.

## Data Availability

Data underlying this article will be made available upon reasonable request to the corresponding author.

## References

[1] IARC Working Group on the Evaluation of Carcinogenic Risks to Humans. Some chemicals that cause tumours of the kidney or urinary bladder in rodents and some other substances. *IARC Monogr Eval Carcinog Risks Hum.* Vol. 73.International Agency for Research on Cancer, 1999. https://publications.iarc.fr/91

[2] The Japan Society for Occupational Health (JSOH) . Recommendation of occupational exposure limits (2023–2024). Environ Occup Health Pract. 2023;5(1):ROEL2023. 10.1539/eohp.ROEL2023 PMC11841793 40059936

[3] Deutsche Forschungsgemeinschaft (DFG) . *List of MAK and BAT Values 2023*. Permanent Senate Commission for the Investigation of Health Hazards of Chemical Compounds in the Work Area, Report 59. Düsseldorf: German Medical Science. Accessed April 29, 2024. 10.34865/mbwl_2023_eng

[4] Nishikawa Y, Okumura T. Determination of nitrilotriacetic acid and ethylenediaminetetraacetic acid in environmental samples as their methyl ester derivatives by gas chromatography-mass spectrometry. J Chromatogr A. 1995;690(1):109–118. 10.1016/0021-9673(94)00919-Z

[5] Ministry of Health, Labour and Welfare (MHLW), Japan . Guidelines for exposure assessment of workers to hazardous substances . Article in Japanese. January 2020. Accessed August 15, 2024. https://www.mhlw.go.jp/content/11305000/000814711.pdf

[6] Hartwig A . MAK Commission. Nitrilotriacetic acid and its sodium salts. MAK value documentation, supplement—translation of the German version from 2020. MAK Collect Occup Health Saf. 2022;7(1):Doc 008. 10.34865/mb13913e7_1ad

[7] Stolzberg RJ, Hume DN. Determination of nitrilotriacetate in environmental water by gas chromatography of the trimethylsilyl ester. Anal Chem. 1977;49(3):374–378. 10.1021/ac50011a013 842850

[8] Randt C, Wittlinger R, Merz W. Analysis of nitrilotriacetic acid (NTA), ethylenediaminetetraacetic acid (EDTA) and diethylenetriaminepentaacetic acid (DTPA) in water, particularly waste water. Fresenius J Anal Chem. 1993;346(6-9):728–731. 10.1007/BF00321281

[9] Raksit A . Gas chromatographic and mass spectrometric analysis of nitrilotriacetic acid in environmental aqueous samples. J AOAC Int. 2002;85(1):50–55. 10.1093/jaoac/85.1.50 11878619

[10] Jiménez JJ . Determination of aminopolycarboxylic acids in river water by solid-phase extraction on activated charcoal cartridges and gas chromatography with mass spectrometric detection. Method performance characteristics and estimation of the uncertainty. Anal Chim Acta. 2013;770:94–102. 10.1016/j.aca.2013.01.060 23498691

[11] Laine P, Matilainen R. Simultaneous determination of DTPA, EDTA, and NTA by UV–visible spectrometry and HPLC. Anal Bioanal Chem. 2005;382(7):1601–1609. 10.1007/s00216-005-3315-0 15971044

[12] Katoh K, Hayama T, Itoyama M, et al. Liquid chromatographic determination of aminopolycarboxylic acids in water samples based on intramolecular excimer-forming fluorescence derivatization. BUNSEKI KAGAKU. 2011;60(1):39–44. 10.2116/bunsekikagaku.60.39

[13] Takeuchi A, Yamamoto S, Narai R, et al. Determination of dimethyl sulfoxide and dimethyl sulfone in urine by gas chromatography–mass spectrometry after preparation using 2,2-dimethoxypropane. Biomed Chromatogr. 2010;24(5):465–471. 10.1002/bmc.1313 19688817

[14] Takeuchi A, Namera A, Sakui N, et al. Direct methyl esterification with 2,2-dimethoxypropane for the simultaneous determination of urinary metabolites of toluene, xylene, styrene, and ethylbenzene by gas chromatography-mass spectrometry. J Occup Health. 2019;61(1):82–90. 10.1002/1348-9585.12026 30698338 PMC6499357

